# The Great Mimicker: Cutaneous Metastatic Melanoma Presenting as a Non-resolving Pleural Effusion

**DOI:** 10.7759/cureus.28320

**Published:** 2022-08-23

**Authors:** Jackeline P Vajta Gomez, Om Parkash, Rebecca Joseph, Janani Arangan, Winston Magno, Monzurul Chowdhury, Carolina Borz-Baba, Consuelito Medrano

**Affiliations:** 1 Internal Medicine, St. Mary's Hospital, Waterbury, USA; 2 Internal Medicine, Icahn School of Medicine, Mount Sinai Hospital, New York, USA; 3 Pathology, St. Mary's Hospital, Waterbury, USA; 4 Nephrology, St. Mary's Hospital, Waterbury, USA; 5 Hematology and Oncology, St. Mary's Hospital, Waterbury, USA

**Keywords:** cancer immunotherapy, non-resolving pleural effusion, metastatic, malignant, cutaneous melanoma

## Abstract

Although melanoma starts as a local disease, it can metastasize to other sites of the body including the lung, brain, liver, and intestines. However, pleural involvement is a rare presentation. Here, we present a case of a 57-year-old man with a history of stage IIA cutaneous melanoma, that relapsed 3 years after cutaneous resection, presenting with a non-resolving pleural effusion. Pleural fluid analysis was consistent with an exudative effusion, and pleural biopsy confirmed metastatic melanoma. The patient was treated with dual therapy of ipilimumab and nivolumab, as per National Comprehensive Cancer Network guidelines, with good response. Thus, we recommend having a high index of clinical suspicion for metastatic pleural melanoma when a patient with a history of cutaneous melanoma presents with a non-resolving pleural effusion.

## Introduction

Melanoma is the fifth most common cancer in the United States [1]. Even though it represents 1% of all skin cancers, it accounts for over 80% of skin cancer deaths [1]. It occurs due to the uncontrolled proliferation of melanocytes [2]. Although melanoma starts as a local malignancy, it can metastasize to other sites of the body, including the lung, brain, liver, bone, and intestines, with the lungs being the most common site of metastasis, accounting for 30% of cases [3,4]. In contrast, malignant pleural effusions account for only 2% of the cases [5]. Here, we present a rare case of cutaneous melanoma stage IIA that metastasized to the lung while presenting as a non-resolving pleural effusion.

## Case presentation

A 57-year-old man presented to the Emergency Department (ED) with a chief complaint of feeling tired and sore for 10 days. Two days prior to presentation, he was short of breath to the point of feeling like he “was drowning in himself,” prompting him to visit an urgent care clinic where he was diagnosed with community-acquired pneumonia and treated with antibiotics. However, his shortness of breath progressed and was associated with pleuritic chest pain, productive cough of white sputum, fatigue, dizziness, and subjective fevers for one week. He denied orthopnea, hemoptysis, sick contacts, travel, or weight loss. He was vaccinated for COVID-19 and never used home oxygen. 

The patient’s past medical and surgical histories included asthma, depression, and a cutaneous melanoma resection of the right upper chest wall with right axillary sentinel lymph nodal resection, 3 years prior to presentation. The cutaneous melanoma had a Breslow thickness of 1.5 mm and Clark's level IV with ulceration and four mitotic figures 4/mm². The axillary sentinel lymph nodes were negative for metastatic involvement. It was classified as Stage IIA melanoma, pT2b, N0, M0. He had a 40 pack-year history of smoking and denied use of alcohol and illicit drugs. Family history was significant for terminal lung cancer in his mother.

Vital signs at the time of ED triage revealed a blood pressure of 141/77 mm/hg, pulse rate of 87 beats/minute, respiratory rate of 20 breaths/minute, temperature of 98.4°F (36.9°C), and oxygen saturation of 97 % on room air. On physical examination, he was in mild respiratory distress. The posterior lung fields were diminished over the right lung base with dullness to percussion, and there was no tenderness to palpation over the chest wall. The heart sounds were normal, and he did not have jugular venous distension or lower extremity edema. Laboratory findings were significant for mild leukocytosis with neutrophilia, low hemoglobin, low hematocrit, elevated D-Dimer, and normal serum lactate dehydrogenase (LDH) and total protein levels (Table 1). 

**Table 1:** Significant labs of the patient on admission. LDH: lactate dehydrogenase

Laboratory Parameters	Patient Values on Admission	Reference Range
WBC (k/µL)	11.7	4.0-10.5
Segmented Neutrophils (%)	66	50
Hemoglobin (g/dL)	13.3	13.5-18.0
Hematocrit (%)	40.9	42.0-54.0
D-Dimer (D-DU ng/mL)	329	<231
Serum LDH (U/L)	212	140-271
Total Protein (g/dL)	6.6	6.0-8.3

**Figure 1:**
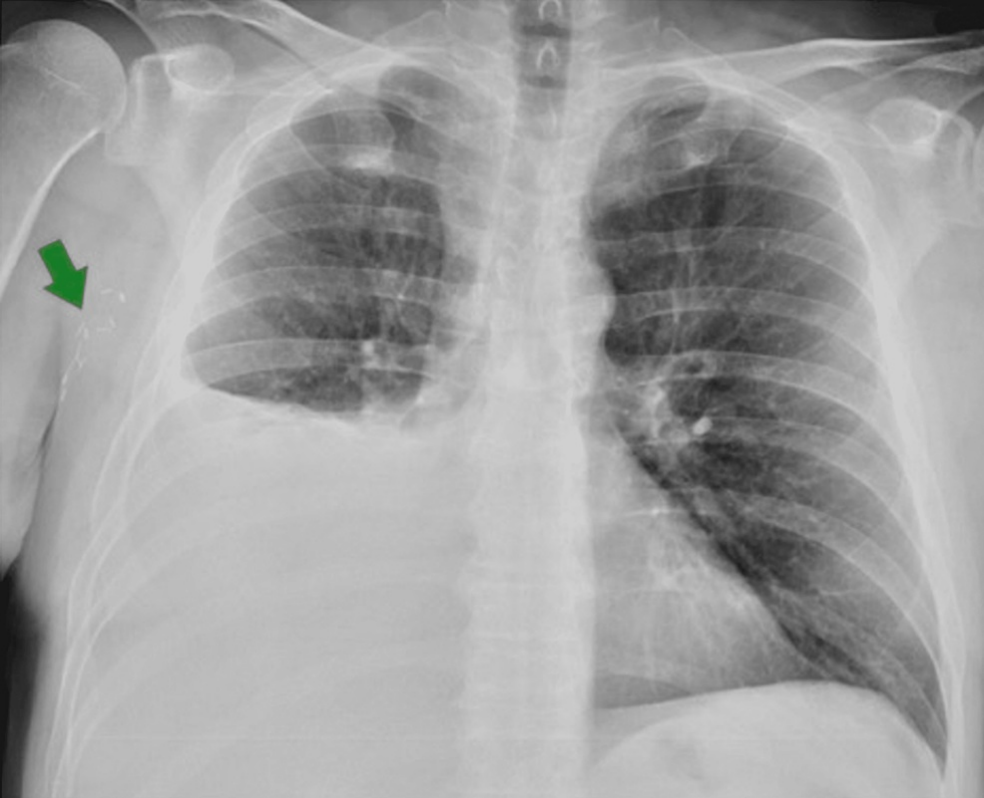
Anteroposterior chest X-ray on presentation. Large right-sided pleural effusion and surgical clips on the right chest wall after cutaneous melanoma resection (green arrow).

The comprehensive metabolic panel, lactate, and brain natriuretic peptide were unremarkable. Chest radiography (CXR) revealed a large right-sided pleural effusion with underlying atelectasis and surgical clips in the axillary region (Figure 1).

**Figure 2:**
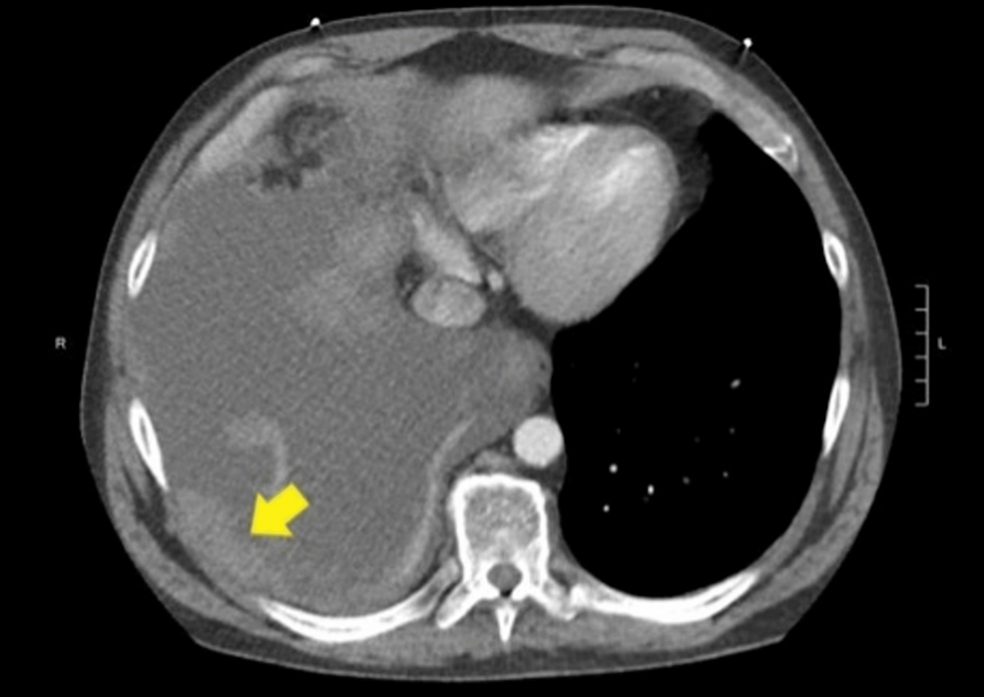
Chest CT with intravenous contrast on presentation. Large right-sided pleural effusion and nodular pleural mass (yellow arrow).

Computer Tomography (CT) of the chest with intravenous contrast confirmed the pleural effusion and revealed a right pleural nodular mass (Figure 2).

No prior CT was available for comparison but a CXR from 3 years prior was normal. The patient underwent diagnostic and therapeutic thoracentesis by interventional radiology (IR) with pleural fluid analysis and cytologic evaluation. Following the removal of 1.5 L of bloody pleural fluid, the patient experienced significant improvement in shortness of breath. Pleural fluid analysis was consistent with an exudative pleural effusion (Table 2).

**Table 2:** Pleural fluid analysis on admission. LDH: lactate dehydrogenase

Pleural Fluid Laboratory Parameters	Patient Values on Admission	Reference Range
WBC (k/µL)	1.587	Not Established
RBC (M/ µL)	0.729	Not Established
Neutrophils (%)	27	Not Established
Eosinophils (%)	26	Not Established
Basophils (%)	0	Not Established
Lymphocytes (%)	31	Not Established
Monocytes (%)	16	Not Established
Pleural LDH (U/L)	433	Not Established
Pleural Protein (g/dL)	4	<3

**Figure 3:**
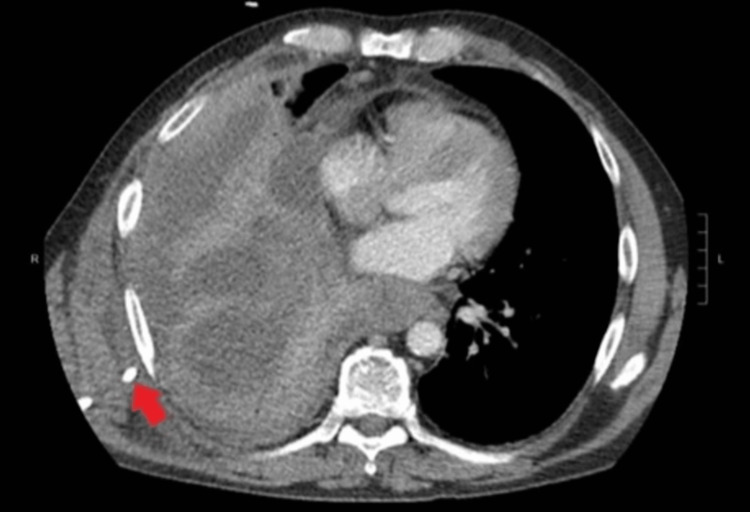
Chest CT with intravenous contrast on the fourth day of admission. Large multiloculated right-sided pleural effusion with associated atelectasis and pleural catheter (red arrow).

Pleural fluid gram stain and culture were negative for any organism or growth. On the second day of admission, the patient developed significant acute hypoxic respiratory distress, requiring oxygen supplementation with 2L via nasal cannula, and a repeat CXR showed re-accumulation of right-sided pleural effusion. A thoracentesis was repeated, removing an additional 0.9 L of similar fluid. On the fourth day, he underwent IR-guided chest tube placement for recurrent pleural effusion, which by that point had become loculated (Figure 3).

**Figure 4:**
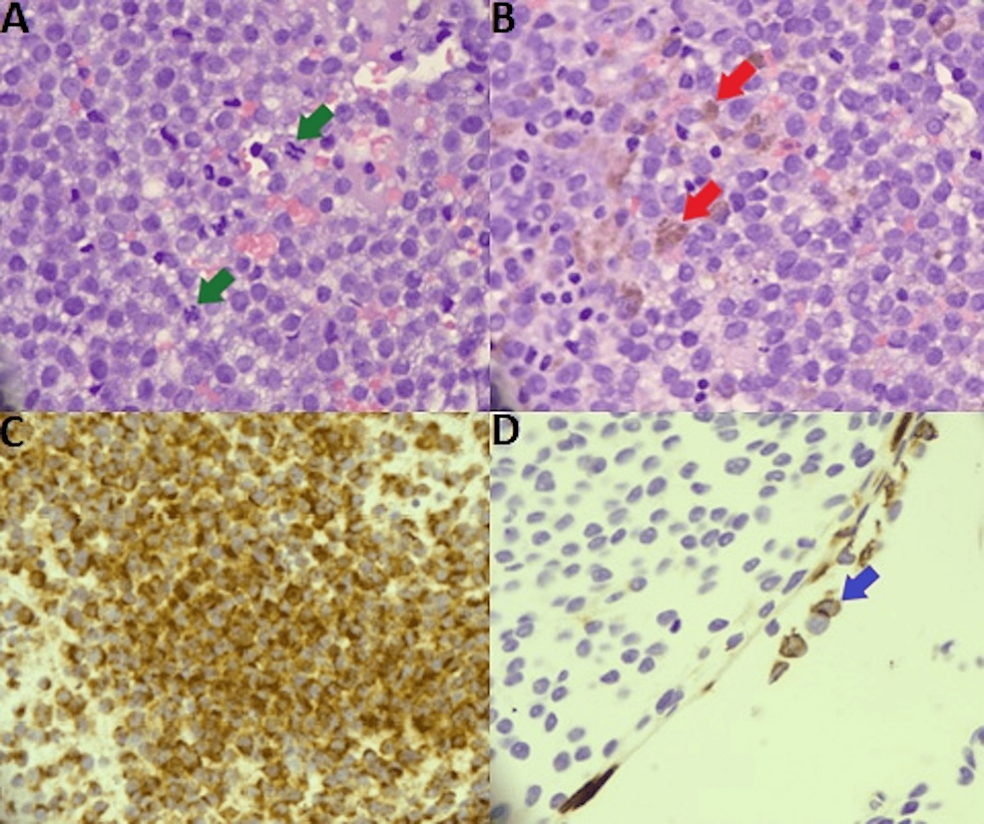
Immunohistochemical markers of pleural mass biopsy. (A) Sheets of melanocytes with occasional mitosis (green arrows), H&Ex400. Round-to-oval cells with round nuclei, some containing pigment with cytoplasm. (B) Some melanocytes with cytoplastic melanin pigment (red arrows), H&Ex400. (C) Melanocytes diffusely positive for HMB-45 and Melan A immunostaining. (D) CK7 D2-40 immunostaining positive with mesothelial cells (blue arrows) and negative within malignant tumor. Abbreviation of stains: Hematoxylin and Eosin (H&E), Monoclonal Antibody Human Melanoma Black-45 (HMB-45), Melanoma Antigen (Melan A), Cytokeratin 7 Monoclonal Antibody D2-40 (CK7 D2-40) Abbreviation of genetic mutations:  V-Raf Murine Sarcoma Viral Oncogene Homolog B1 (BRAV) V600E Mutation. Phosphatase and TENsin Homolog On Chromosome 10 (PTEN) Mutation

This was managed with intrapleural fibrinolytic therapy with a tissue plasminogen activator (tPA). On the eighth day of admission, thoracic surgery was consulted for the procurement of additional tissue. The patient underwent video-assisted thoracoscopy surgery (VATS) with a biopsy of the right lung pleural mass. Pathology and tissue samples were reviewed, confirming a diagnosis of metastatic melanoma per positive immunochemical markers including HMB-45, Sox10, Melan-A, and S100 (Figure 4), along with positive BRAF(V600E) mutation with PTEN deletion.

**Figure 5:**
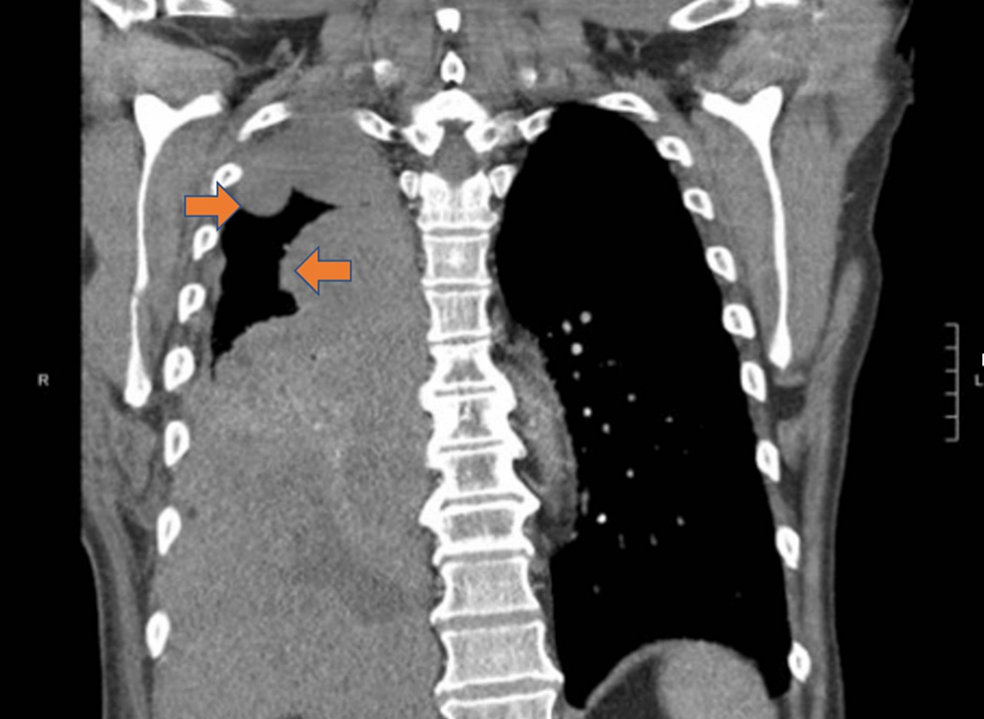
Chest CT with intravenous contrast on readmission two weeks after initial discharge. Extensive enlargement of right pleural masses (orange arrows), consistent with progression of pleural malignancy.

The patient was transferred to the oncology floor for initiation of ipilimumab and nivolumab therapy. On the seventeenth day of hospitalization, oxygen supplementation was successfully weaned off. He was discharged home with narcotics and oncology follow-up. Unfortunately, he was readmitted multiple times due to thoracic pain, acute hypoxic respiratory failure with recurrent pleural effusion, and extension of pleural masses on chest CT (Figure 5).

**Figure 6:**
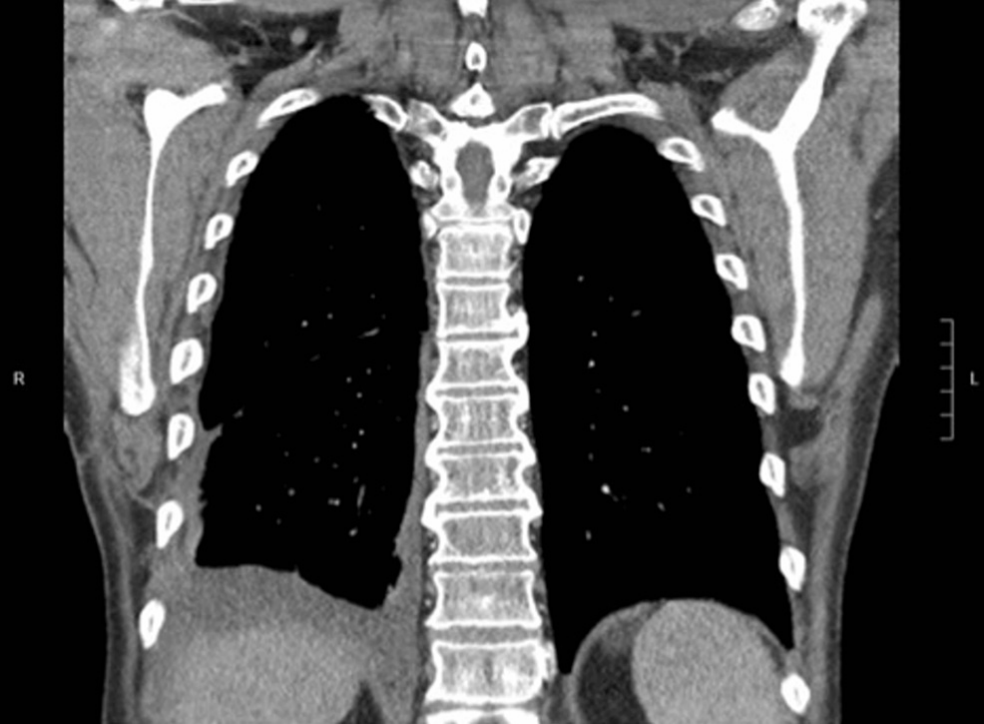
Chest CT with intravenous contrast at nine weeks after initial discharge, eight weeks after treatment. Significantly less pronounced pleural masses with small right-sided pleural effusion.

Thoracic pain was significantly improved after nerve block. The therapeutic regimen of ipilimumab and nivolumab was continued despite chest CT findings concerning for disease progression. Subsequent chest CT revealed decreased tumor burden correlating with proper treatment response (Figure 6).

The patient tolerated immunotherapy well. At the end of four cycles of the treatment, he developed a mild rash, grade 2 per Common Terminology Criteria of Adverse Effects. This is typical of immunotherapy and was managed with topical steroids.

## Discussion

Most cases of malignant melanoma have cutaneous spread, but when present, distant metastases have a poor prognosis. Most metastatic relapses occur within three years, and late metastases can occur even after ten years [6,7]. Pleural involvement, although rare, can present as pleural thickening, mimicking mesothelioma, or pleural effusion, as in the case of our patient [8]. 

A new large pleural effusion generates a broad differential diagnosis; and the diagnostic evaluation should follow a standard algorithm including inspection of the gross appearance of the fluid, laboratory workup to help differentiate exudative from transudative fluid, and finally cytologic analysis. In the case of melanoma, the presence of melanocytes may confer the pleural effusion a black color as previously described in literature [9]. As with other malignant effusions, we expect the fluid to be exudative rather than transudative. It is estimated that 60% of the time, a malignant pleural effusion will be diagnosed on pleural fluid cytology [10]. Moreover, metastatic spread to the lung develops on an average of about 3.1 years later in patients with resected stage I melanoma [11]. The pleural effusion can be unilateral or bilateral, generally small, and can be associated with pulmonary nodules and adenopathy [11]. In the case of our patient, the effusion was large, serosanguinous in color, and exudative in nature. Although the fluid cytology identified some melanocytes in the sample, a repeated diagnostic thoracentesis with pleural fluid cytology had to be performed since the initial sample was unsatisfactory for appropriate melanocyte staining. A temporary pleural drainage catheter was in place to aid in fluid drainage. Pleural biopsy confirmed metastatic melanoma via melanocyte cell HMB-45 and Melanin A immunostaining and BRAF(V600E) mutation. 

Surgical treatment with wide local excision and sentinel node biopsy represents the standard of care in cutaneous melanoma. Current surveillance guidelines as per National Comprehensive Cancer Network, recommend that all patients be followed up with a history and physical exam with an emphasis of nodes and skin. Routine surveillance imaging is not recommended for stages I-IIA, whereas it is suggested for stages IIb-IV. Since most relapses occur within 3 to 5 years, routine imaging is not recommended after this period [12,13]. The advantages of including surveillance imaging are the early detection of asymptomatic recurrent disease and potential identification of an additional malignancy [13]. The prospective harm in using surveillance imaging is the low probability of detecting a small size recurrence (< 5 mm) by CT or positron emission tomography, or the risk of false positive results associated with significant anxiety [13]. 

Our patient was initially scheduled for surveillance 4 months after excision with surgical oncology. However, he was lost to follow-up due to the COVID-19 pandemic and was admitted with symptoms 3 years later related to pleural metastasis. The CT chest performed in our patient’s case revealed a large pleural effusion and multiple pleural nodules, which are rarely reported in the literature. 

Treatment for stage IV melanoma includes a combination therapy with nivolumab and ipilimumab as the first-line treatment for metastasis. This combination has radically changed the clinical practice and outcome of metastatic melanoma, through its sustained effects. For patients with BRAF-activating mutation, BRAF/MEK inhibitor therapy can be used in those unable to tolerate immune checkpoint inhibitors, or those necessitating a faster response as immune checkpoint inhibitors can have a delayed response [14]. 

Another genetic aberration that is frequently found in metastatic melanoma is the deletion of chromosome 10q. Although the prognostic significance of this marker and the potential benefits of targeted therapy are being investigated, this mutation deserves importance as it contains the tumor suppressor gene PTEN, responsible for promoting tumor evasion, and whose deletion or mutation is commonly associated with melanoma spread [15,16]. Our patient had this deletion, placing him at increased risk of melanoma relapse.

Prior literature has shown that long-term overall survival at 5 years was higher in patients who were treated with nivolumab plus ipilimumab or nivolumab as compared with ipilimumab alone with a hazard ratio for death of 0.52 (95% CI, 0.42 to 0.64; P<0.001) for nivolumab plus ipilimumab vs. ipilimumab, 0.63 (95% CI, 0.52 to 0.76; P<0.001) for nivolumab vs. ipilimumab, and 0.83 (95% CI, 0.67 to 1.03) for nivolumab plus ipilimumab vs. nivolumab [17]. Our patient received four cycles of immunotherapy with nivolumab 1mg/kg and ipilimumab 3mg/kg monthly, then transitioned to nivolumab maintenance.

Although he was readmitted multiple times due to acute hypoxic respiratory failure which was attributed to the delayed response of immunotherapy, manifesting as tumor pseudo-progression and transient worsening of symptoms; subsequent imaging with chest CT revealed decreased tumor burden correlating with proper treatment response. 

## Conclusions

This is an atypical presentation of a cutaneous melanoma metastasizing to the lung pleura in 3 years after cutaneous resection and presenting as a non-resolving pleural effusion. Cutaneous melanoma can metastasize and relapse to many places within the body, but most commonly to the lungs. This case highlights the importance of having a high index of clinical suspicion of metastatic relapse in patients with early-stage melanoma including stage IIA, to improve treatment outcomes and morbidity. Additionally, immunotherapy can have a delayed response that can manifest as a pseudo-progression of tumor on imaging, symptoms, or physical exam before seeing a clinical response.
